# Declining trends in the rates of assisted injecting: a prospective cohort study

**DOI:** 10.1186/s12954-016-0092-3

**Published:** 2016-01-27

**Authors:** Jeanette Somlak Pedersen, Huiru Dong, Will Small, Evan Wood, Paul Nguyen, Thomas Kerr, Kanna Hayashi

**Affiliations:** Cumming School of Medicine, University of Calgary, 3330 Hospital Drive NW, Calgary, AB T2N 4N1, Canada; British Columbia Centre for Excellence in HIV/AIDS, St. Paul’s Hospital, 608-1081 Burrard Street, Vancouver, BC V6Z 1Y6 Canada; Faculty of Health Sciences, Simon Fraser University, 8888 University Drive, Burnaby, BC 15A 1S6 Canada; Department of Medicine, University of British Columbia, St. Paul’s Hospital, 608-1081 Burrard Street, Vancouver, BC V6Z 1Y6 Canada; B.C. Centre for Excellence in HIV/AIDS, University of British Columbia, St. Paul’s Hospital, 608-1081 Burrard Street, Vancouver, BC V6Z 1Y6 Canada

**Keywords:** Assisted injecting, Injection drug use, Harm reduction, Vancouver

## Abstract

**Background:**

Assisted injecting has been associated with increased risk of blood-borne infections, overdose, and other harms among people who inject drugs (PWID), particularly women. Given the changing availability of relevant harm reduction interventions in Vancouver, Canada, in recent years, we conducted a gender-based analysis to examine changes in rates and correlates of assisted injecting over time among active PWID.

**Methods:**

Using data from a prospective cohort of PWID in Vancouver, we employed gender-stratified multivariable generalized estimating equations to examine trends in assisted injecting and identify the correlates during two periods: June 2006–November 2009 and December 2009–May 2014.

**Results:**

Among 1119 participants, 376 (33.6 %) were females. Rates of assisted injecting declined between 2006 and 2014 among males (21.9 to 13.8 %) and females (37.0 to 25.6 %). In multivariable analyses, calendar year of interview also remained independently and negatively associated with assisted injecting among males (adjusted odds ratio [AOR] 0.95, 95 % confidence interval [CI] 0.92–0.99) and females (AOR 0.93, 95 % CI 0.89–0.97). Syringe borrowing remained independently associated with assisted injecting throughout the study period among females (AOR 1.53, 95 % CI 1.10–2.11 during 2006–2009; AOR 2.15, 95 % CI 1.24–3.74 during 2009–2014) and during 2009–2014 among males (AOR 1.88, 95 % CI 1.02–3.48).

**Conclusions:**

Our findings demonstrate assisted injecting has significantly decreased for both males and females over the past decade. Nevertheless, rates of assisted injecting remain high, especially among women, and are associated with high-risk behavior, indicating a need to provide safer assisted injecting services to these vulnerable sub-populations of PWID.

## Background

Injection drug use is a major public health issue associated with significant health and social consequences. People who require assistance injecting have been shown to be a particularly vulnerable subgroup of people who inject drugs (PWID) [1, 2]. Assisted injection, which refers to manual administration of an injection to another person [3], is common among PWID, with a 1996–2002 study estimating that 41 % of PWID in a large Canadian city reported assisted injecting within the previous 6 months [1].

Assisted injecting is a major risk factor for many negative health outcomes. As those who provide assistance injecting often use the same syringe between two individuals, there is an independent association between requiring help injecting and syringe sharing—a well-established risk behavior for blood-borne infections [4, 5]. Research has documented that assisted injecting is strongly associated with HIV [1, 6, 7], hepatitis C [8], and cutaneous injection-related infections [9]. One Canadian study found a twofold higher risk of HIV infection among those requiring assistance injecting [1]. The negative health consequences of assisted injecting are not only limited to infections but also include non-fatal overdoses [10] and vulnerability to various forms of violence (e.g., robbery) [11].

Previous research has indicated that female PWID may be more likely than males to require assistance with injecting [1, 2, 4, 12]. The higher rates of assisted injecting among females may be due to social and interpersonal dynamics where males often control the use of drugs within relationships [1, 6, 11, 13, 14]. Consequently, females are commonly injected by males [11], and in turn, many women report requiring assistance with injecting due to a lack of knowledge on how to inject themselves [2]. Although the reasons for needing assistance with injecting may be highly gendered, common reasons for assisted injecting include a lack of viable veins, reliance upon jugular injection, being in withdrawal, and a lack of knowledge of how to inject [2, 4].

In Vancouver, Canada, some harm reduction strategies have been implemented over the past decade to address the harms associated with assisted injecting. In September 2003, a medically supervised injection facility (SIF) opened in the city’s Downtown Eastside neighborhood, an area with high levels of injection drug use and HIV infection [15]. There, healthcare staff provides education on safer injection techniques [16]; however, the laws governing the SIF do not allow healthcare staff and peers to perform manual assistance with injecting, constituting a significant barrier for some PWID who are unable to self-inject [11]. In response, in 2005, the Vancouver Area Network of Drug Users (VANDU), a local drug user organization, began operating the Injection Support Team (IST) whereby trained peer volunteers walked the streets of the Downtown Eastside providing education, support, and assistance with injections [3]. However, it ceased operating in 2009 due to a lack of funding. Subsequently, between 2011 and 2013, a peer-run unsanctioned SIF was operated by VANDU in the Downtown Eastside, where trained peer volunteers provided assisted injections under a strict harm reduction policy ensuring safe injection practices; however, compared to IST, it was on a smaller scale [11]. In many settings around the world, except for some European countries, liabilities related to providing assistance with injecting of illicit drugs pose challenges for developing harm reduction interventions other than provision of safer injection education [17].

While a recent study has suggested a declining impact of assisted injecting on HIV incidence in Vancouver [18], little is known about changes in the prevalence and the associated harms of assisted injection over time in this setting, and how they may differ across the genders. Therefore, we conducted a gender-based analysis among PWID in Vancouver to examine trends in the rates of requiring assisted injecting over time. In sub-analyses, we also examined changes in the correlates of and reasons for requiring assisted injecting over time.

## Methods

### Study design

Data were collected from the Vancouver Injection Drug Users Study (VIDUS), a prospective cohort study of PWID in Vancouver, Canada. Recruitment of VIDUS participants began in the Downtown Eastside neighborhood in May 1996. The VIDUS cohort has been described in detail previously [19]. In brief, eligibility criteria include being aged ≥18 years, injecting illicit drugs at least once in the preceding month, living in greater Vancouver area, and providing informed consent. At baseline and subsequent semi-annual follow-up interviews, participants complete an interviewer-administered questionnaire, which includes items on sociodemographics, drug use patterns, and other characteristics and exposures. At each visit, participants provide blood samples to test for HIV and hepatitis C and receive CAD $30 in monetary compensation. The VIDUS cohort has been approved by the University of British Columbia/Providence Healthcare Research Ethics Board.

For the present analyses, participants were eligible if they completed the baseline assessment between December 1, 2005 and May 31, 2014. The sample was further restricted to those who reported having injected drugs in the previous 6 months for each subsequent follow-up.

### Study variables

The main outcome of interest was assisted injecting in the past 6 months, defined as responding “yes” to the question: “In the last 6 months, did someone help you inject?” The primary explanatory variable was calendar year of interview (per year later). Based on the literature [2, 4, 20], we also selected a range of secondary explanatory variables that we hypothesized to be associated with assisted injecting. Binary variables (yes vs. no) included the following: Caucasian ancestry; not completing high school education; currently in a stable relationship; homelessness; sex work, defined as exchanging sex for gifts, food, shelter, clothes, etc.; incarceration; and being a victim of violence; ≥daily heroin injection; ≥daily cocaine injection; ≥daily crystal methamphetamine injection; ≥daily prescription opioid injection; any public injection drug use; syringe borrowing; non-fatal overdose; and ever learned safe injection technique by a healthcare provider assessed at baseline. A continuous variable included years injecting (per 10 years longer). Time-varying sociodemographic and drug use variables referred to the previous 6 months unless otherwise indicated.

### Statistical analyses

All analyses were stratified by gender. First, we examined the baseline sample characteristics stratified by reports of assisted injecting using Pearson’s chi-squared test (for binary variables) and Wilcoxon rank-sum test (for continuous variables). Fisher’s exact test was used when one or more of the cells contained expected values less than or equal to five. We also plotted proportions of participants reporting assisted injecting over the calendar year of interview.

Since the analyses of assisted injecting included serial measures for each participant, we used generalized estimating equations (GEE) with logit link, which provided standard errors adjusted by multiple observations per person using an exchangeable correlation structure. To examine the relationship between the calendar year of interview and assisted injecting, we fit multivariable GEE models using a conservative confounding model selection approach [21]. We included all variables that were associated with assisted injecting in unadjusted analyses at *p* < 0.10 in a full multivariable model, and used a stepwise approach to fit a series of reduced models. After comparing the value of the coefficient of the calendar year of interview in each reduced model, we dropped the secondary variable associated with the smallest relative change. We continued this iterative process until the minimum change exceeded 5 %.

Because each participant contributed a different number of study visits, we further conducted a sensitivity analysis using independence estimating equations (IEE) with the same confounding model selection approach. IEE examined the relationship between the calendar year of interview and assisted injecting, allowing for potential informative cluster size in the analysis [22].

In a sub-analysis to identify changes in the correlates of assisted injecting over time, we divided the study period into two sub-periods (June 2006–November 2009 and December 2009–May 2014) based on the VANDU IST operation period and fit multivariable models separately. Both periods included the same set of variables described above with the only difference being the presence/absence of a variable assessing the use of the VANDU IST in the previous 6 months (yes vs*.* no). We determined the covariates to be included in the final multivariable models using an a priori-defined model-building procedure. The procedure started with all covariates that were associated with assisted injecting at the level of *p* < 0.10 in unadjusted analyses, and proceeded using a backward selection process while two variables (i.e., years injecting and accessing VANDU IST) were forced to remain in the models. The final multivariable models with the lowest quasi-likelihood under the independence model criterion value were selected [23].

Lastly, we also examined changes in the reasons for requiring assisted injecting over time. Since the question asking about reasons for assisted injecting was removed from the questionnaire during some periods in the follow-up, only the data collected during the first and last 18 months of the study period (i.e., December 2005–May 2007 and December 2012–May 2014) were available for this analysis. We used Pearson’s chi-squared test (or Fisher’s exact test when one or more of the cells contained expected values less than or equal to five) to compare the reasons for assisted injecting between the two 18-month periods. All *p* values were two-sided. All statistical analyses were performed using the SAS software version 9.4 (SAS, Cary, NC).

## Results

### Summary statistics

**Fig. 1:**
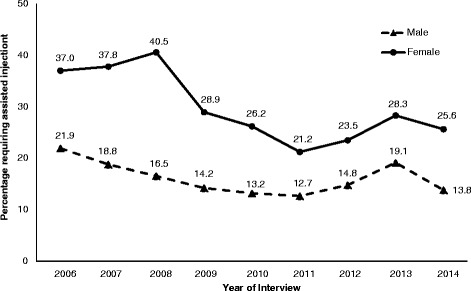
Rates of requiring assistance with injecting among PWID by year of interview. *PWID* people who inject drugs

A total of 1119 participants, including 376 (33.6 %) female PWID, were included in this study. Of these, 151 (20.3 %) males and 127 (33.8 %) females reported requiring assisted injecting in the previous 6 months at baseline. Table 1 shows the baseline sample characteristics stratified by requiring assisted injecting in the previous 6 months. As can be seen in Fig. 1, rates of requiring assistance with injecting have decreased for both males and females over time. In 2006, 37.0 % of females reported assisted injecting in the previous 6 months compared to 25.6 % in 2014. Similarly, in 2006, 21.9 % of males reported assisted injecting in the previous 6 months compared to 13.8 % in 2014.

**Table 1:** Baseline sample characteristics stratified by requiring assistance with injecting in the previous 6 months among PWID in Vancouver, Canada (*n* = 1119)

Characteristic	Males (*n* = 743)			Females (*n* = 376)		
	Requiring assistance with injecting^a^		*p* value	Requiring assistance with injecting^a^		*p* value
	Yes (%)	No (%)		Yes (%)	No (%)	
	151 (20.3)	592 (79.7)		127 (33.8)	249 (66.2)	
Demographic and social characteristics						
Age (median, IQR)	43 (36–48)	42 (35–48)	0.596	36 (28–43)	37 (29–45)	0.605
Caucasian	100 (66.2)	409 (69.1)	0.499	70 (55.1)	125 (50.2)	0.367
<High school diploma	78 (51.7)	249 (42.1)	0.039	65 (51.2)	141 (56.6)	0.368
In a stable relationship	40 (26.5)	148 (25.0)	0.683	55 (43.3)	96 (38.6)	0.390
Homeless^a^	63 (41.7)	230 (38.9)	0.519	57 (44.9)	86 (34.5)	0.051
Sex work^a^	7 (4.6)	11 (1.9)	0.069	54 (42.5)	102 (41.0)	0.822
Incarcerated^a^	29 (19.2)	115 (19.4)	0.951	28 (22.1)	33 (13.3)	0.031
Victim of violence^a^	50 (33.1)	137 (23.1)	0.013	43 (33.9)	51 (20.5)	0.007
Drug use-related characteristics						
Years injecting (median, IQR)	20 (8–30)	19 (12–31)	0.347	12 (8–24)	16 (10–24)	0.044
≥Daily heroin injection^a^	52 (34.4)	187 (31.6)	0.504	62 (48.8)	86 (34.5)	0.007
≥Daily cocaine injection^a^	20 (13.3)	52 (8.8)	0.100	18 (14.2)	27 (10.8)	0.347
≥Daily crystal meth injection^a^	14 (9.3)	24 (4.1)	0.009	8 (6.3)	9 (3.6)	0.229
≥Daily PO injection^a^	8 (5.3)	47 (7.9)	0.269	7 (5.5)	12 (4.8)	0.772
Injecting in public^a^	77 (51.0)	242 (40.9)	0.021	67 (52.8)	92 (37.0)	0.003
Syringe borrowing^a^	15 (9.9)	56 (9.5)	0.860	22 (17.3)	21 (8.4)	0.011
Non-fatal overdose^a^	18 (11.9)	36 (6.1)	0.014	14 (11.0)	23 (9.2)	0.608
Accessed VANDU Injection Support Team^a^	26 (17.2)	67 (11.3)	0.014	17 (13.4)	26 (10.4)	0.574
Ever learned safe injection technique by healthcare provider	34 (22.5)	124 (21.0)	0.932	37 (29.1)	45 (18.1)	0.026

*PWID* people who inject drugs, *IQR* interquartile range, *PO* prescription opioid, *VANDU* Vancouver Area Network of Drug Users

^a^Activities in the previous 6 months

### Primary analyses

The declining trends in assisted injecting were consistent with the results of the multivariable GEE analyses. As shown in Table 2, after an extensive confounder adjustment, the calendar year of interview remained independently and negatively associated with assisted injecting among both males (adjusted odds ratio [AOR] 0.95, 95 % confidence interval [CI] 0.92–0.99) and females (AOR 0.93, 95 % CI 0.89–0.97).

**Table 2:** Univariable and multivariable GEE analyses of factors associated with requiring assistance with injecting among PWID in Vancouver, Canada (*n* = 1119)

Characteristic	Males		Females	
	Unadjusted OR	Adjusted OR	Unadjusted OR	Adjusted OR
	(95 % CI)	(95 % CI)	(95 % CI)	(95 % CI)
Interview year				
(per year later)	0.95 (0.92–0.99)	0.95 (0.92–0.99)	0.90 (0.87–0.94)	0.93 (0.89–0.97)
Ethnicity				
(Caucasian vs*.* other)	1.09 (0.80–1.49)		1.29 (0.91–1.81)	
Education				
(<high school diploma vs*.* ≥high school diploma)	1.14 (0.85–1.53)		0.95 (0.66–1.35)	
Currently in a stable relationship				
(yes vs*.* no)	1.20 (0.99–1.47)		1.05 (0.86–1.28)	
Homeless^a^				
(yes vs*.* no)	1.10 (0.91–1.32)		1.53 (1.23–1.90)	
Sex work^a^				
(yes vs*.* no)	2.67 (1.48–4.80)	2.17 (1.18–3.99)	1.48 (1.21–1.82)	
Incarcerated^a^				
(yes vs*.* no)	1.08 (0.86–1.34)		1.57 (1.19–2.09)	
Victim of violence^a^				
(yes vs*.* no)	1.35 (1.13–1.62)	1.24 (1.03–1.48)	1.56 (1.26–1.93)	1.31 (1.06–1.62)
Years since first injection				
(per 10 years longer)	0.85 (0.75–0.97)		0.60 (0.49–0.73)	0.80 (0.65–0.98)
Heroin injection^a^				
(≥daily vs*.* <daily)	1.37 (1.15–1.63)		1.53 (1.23–1.90)	
Cocaine injection^a^				
(≥daily vs*.* <daily)	1.56 (1.23–1.98)		0.92 (0.70–1.22)	
Crystal meth injection^a^				
(≥daily vs*.* <daily)	2.11 (1.49–2.99)	2.20 (1.54–3.14)	0.99 (0.53–1.87)	
PO injection^a^				
(≥daily vs*.* <daily)	1.01 (0.77–1.33)		0.98 (0.65–1.48)	
Injecting in public^a^				
(yes vs*.* no)	1.67 (1.41–1.97)	1.56 (1.31–1.85)	1.90 (1.57–2.30)	1.59 (1.29–1.96)
Syringe borrowing^a^				
(yes vs*.* no)	1.38 (1.07–1.77)		1.96 (1.54–2.49)	1.47 (1.14–1.91)
Non-fatal overdose^a^				
(yes vs*.* no)	1.56 (1.20–2.03)		1.23 (0.89–1.70)	
Ever learned safe injection technique by healthcare provider				
(yes vs*.* no)	1.05 (0.75–1.47)		1.93 (1.26–2.96)	1.80 (1.16–2.79)

*PWID* people who inject drugs, *PO* prescription opioid, *OR* odds ratio, *CI* confidence interval

^a^Activities/events in the past 6 months

Consistent with the GEE analyses, the sensitivity analysis using IEE also found declining trends in assisted injecting among both males (for the calendar year of interview, AOR 0.95, 95 % CI 0.91–0.99) and females (AOR 0.93, 95 % CI 0.88–0.98).

### Sub-analyses

In the multivariable GEE analyses of factors associated with assisted injecting during two time periods, June 2006–November 2009 and December 2009–May 2014, there were some persistent and changing correlates of assisted injecting between the genders as well as between the two time periods. Among males, during the first time period, sex work (AOR 3.75, 95 % CI 1.68–8.33) and injecting in public (AOR 1.41, 95 % CI 1.07–1.85) were independently associated with assisted injecting. During the second time period, daily crystal meth injection (AOR 2.98, 95 % CI 1.93–4.61), injecting in public (AOR 2.00, 95 % CI 1.46–2.73), and syringe borrowing (AOR 1.88, 95 % CI 1.02–3.48) were independently associated with assisted injecting. Among females, during the first time period, sex work (AOR 1.45, 95 % CI 1.08–1.93), injecting in public (AOR 1.97, 95 % CI 1.48–2.62), and syringe borrowing (AOR 1.53, 95 % CI 1.10–2.11) were independently associated with requiring assistance with injecting. During the second time period, daily heroin injection (AOR 1.63, 95 % CI 1.17–2.28), injecting in public (AOR 1.45, 95 % CI 1.08–1.96), and syringe borrowing (AOR 2.15, 95 % CI 1.24–3.74) were independently associated with assisted injecting.

Table 3 shows reasons for assisted injecting during the first and last 18 months of the study period, December 2005–May 2007 and December 2012–May 2014. As shown, the three most common reasons for males requiring assistance with injecting during the first 18 months included having bad veins/no veins (40.4 %), jugular injection (21.2 %), and a lack of injection technique (20.5 %). In contrast, during the last 18 months, the ranking slightly changed, including jugular injection (33.3 %), bad veins/no veins (30.8 %), being anxious/dope sick (14.1 %), and vision or other disability (14.1 %). Significantly more males reported jugular injection to be a reason for assisted injecting in the second compared to first period (*p* = 0.045). Among females, the most common reasons during the first 18 months included having bad veins/no veins (41.7 %), jugular injection (37.4 %), and a lack of injection technique (16.5 %). Again, the ranking during the last 18 months slightly changed and included jugular injection (45.0 %), bad veins/no veins (30.0 %), and being anxious/dope sick (21.7 %). Significantly more females reported being anxious/dope sick to be a reason for assisted injecting in the second compared to first time period (*p* = 0.016).

**Table 3:** Reasons for assisted injecting among PWID during two time periods (December 2005–May 2007 and December 2012–May 2014)

Reason for needing help	Males (*n* = 229)			Females (*n* = 175)		
	December 2005–May 2007 (%)	December 2012–May 2014 (%)	*p* value	December 2005–May 2007 (%)	December 2012–May 2014 (%)	*p* value
	151 (65.9)	78 (34.1)		115 (65.7)	60 (34.3)	
Lack of injection technique	31 (20.5)	8 (10.3)	0.050	19 (16.5)	2 (3.3)	0.013
Bad veins/no veins	61 (40.4)	24 (30.8)	0.153	48 (41.7)	18 (30.0)	0.128
Anxious/dope sick	22 (14.6)	11 (14.1)	0.924	10 (8.7)	13 (21.7)	0.016
Jugular injection	32 (21.2)	26 (33.3)	0.045	43 (37.4)	27 (45.0)	0.329
Vision or other disability	24 (15.9)	11 (14.1)	0.721	17 (14.8)	7 (11.7)	0.570
Other reasons	7 (4.6)	9 (11.5)	0.052	4 (3.5)	1 (1.7)	0.662

*PWID* people who inject drugs

## Discussion

In our study, rates of assisted injecting in the previous 6 months have declined between 2006 and 2014 for both males (21.9 to 13.8 %) and females (37.0 to 25.6 %). The declining trends were consistent with the results of multivariable GEE analyses in which, after extensive confounder adjustments, the calendar year of interview remained independently and negatively associated with assisted injecting among both genders. Further, syringe borrowing remained independently and positively associated with assisted injecting throughout the study period among females and in more recent years among males. In the last 18-month period (between December 2012 and May 2014), the top three commonly reported reasons for assisted injecting were similar between males and females and included jugular injection, bad veins/no veins, and being anxious/dope sick. For both genders, proportions of participants reporting a lack of injection technique as a reason for assisted injecting significantly decreased in more recent years.

We have demonstrated a significant decline in the rates of assisted injecting for both male and female PWID over time even after adjusting for a range of potential confounders. To our knowledge, this is the first study examining trends in rates of assisted injecting over time. The declining rates among PWID in this setting are encouraging and may be related to increased awareness of the risks associated with assisted injecting due to improved access to harm reduction information and interventions. Although the present study did not directly examine whether access to such interventions helped PWID stop assisted injecting, our findings that proportions of PWID reporting “a lack of injection technique” as a reason for requiring assisted injecting significantly decreased among both genders in more recent years suggest that this might have been the case for some PWID. However, it is important to note that rates of assisted injecting in recent years continue to be high despite the decline demonstrated in this study. This is particularly true for females, which is consistent with previous studies demonstrating higher rates of assisted injecting among females than males [1, 2, 4]. Research has highlighted the social and structural context within which assisted injecting commonly occurs, including the role of social and interpersonal dynamics as well as social rules of providing money or drugs in exchange for assistance [20]. For females, assisted injecting is often a feature of intimate or romantic relationships where male partners commonly control the drugs to be injected [14, 20]. This has been argued to further subordinate women within drug scenes and increasing their vulnerability to negative health consequences such as blood-borne infections [11, 24, 25]. Thus, we recommend that future interventions examine ways of addressing the gender disparity in rates of assisting injecting, including consideration of the social and structural context.

Our findings support the existing literature by demonstrating that syringe borrowing has continued to be common among PWID requiring assistance with injecting, particularly among women. In the context of intimate relationships, qualitative findings have demonstrated that women are often injected by controlling “boyfriends” [11]. This increases the likelihood that boyfriends will inject themselves first and then using the same syringe on them after. In other words, gendered power dynamics play an important role in increasing the risk of syringe borrowing among women who rely on assisted injecting. A concerning finding of our study is that the association between assisted injecting and syringe borrowing appears to have strengthened in recent years (compared to the first time period studied). We hypothesize that this may be due to a lack of specific interventions targeting syringe sharing among those who require assistance with injecting.

Our study demonstrates some noteworthy trends in drug use patterns associated with assisted injecting over time. Consistent with previous studies [26], injecting in public was identified as a persistent correlate of assisted injecting for both males and females throughout the study period. This is concerning as injecting safely in public is often challenged by unhygienic locations, interruption, violence, and police presence [26–28], all of which increase risks to the harmful effects of assisted injecting. Our study also highlights some changing trends in drug use patterns. Daily crystal meth injection was identified as an independent correlate of assisted injecting among males in recent years. There has been an increasing trend in crystal meth injections in Vancouver in recent years [29]. People who inject drugs of a short half-life, such as crystal meth, tend to inject many times a day and are vulnerable to vascular damage and blood-borne disease acquisition [30]. They may also subsequently progress to injecting in more risky ways, such as the jugular vein injecting [30], which is associated with requiring assistance with injecting [31]. Our finding that jugular injection as a reason for assisted injecting has increased among males in recent years supports this hypothesis, although loss of access to peripheral veins as the VIDUS cohort ages is another possible explanation for transitioning to jugular injections. Among females, daily heroin injection was identified as an independent correlate of assisted injecting in recent years, and anxiousness and dope sickness as a reason for requiring assistance with injecting has also increased in recent years among females. Dope sickness is common in people withdrawing from heroin; thus, the increase in anxiousness and dope sickness is consistent with the emergence of daily heroin injection as an independent correlate of assisted injecting among females. However, our study was unable to examine why different drugs were identified as independent correlates between the genders. Further qualitative research is needed to explore this issue.

The findings presented in this study have a number of important implications for policy and research. We agree with previous researchers [1, 11, 12] that allowing assisted injecting at SIFs would result in potential health benefits for PWID. Not only would it likely decrease the overall risky rate of assisted injecting (e.g., through extending the reach of the SIFs to a large subgroup of PWID, who require manual assistance with injecting and are at a heightened risk of harms associated with assisted injecting due to the social and structural factors), allowing assisted injecting at SIFs would also decrease the number of PWID who inject in public outdoor spaces thereby decreasing their vulnerability to harms associated with public outdoor assisted injecting [12]. Furthermore, permitting assisted injecting at SIFs would also provide a significant sub-population of PWID with access to clean syringes, thereby reducing their risk of blood-borne infections. Indeed, a previous evaluation of an unsanctioned peer-run SIF that allowed assisted injecting demonstrated the feasibility and potential benefits of this approach [32], indicating that it reshaped the social and structural contexts surrounding assisted injection to reduce exposure to HIV risks and drug scene violence. Secondly, previous studies have also demonstrated that trained peer educators can provide helpful education and safer injections to PWID who require assistance injecting [3, 11]. Therefore, access to safe peer-driven assisted injecting services both within SIFs as well as in the community, such as the VANDU IST, should be promoted.

This study has some limitations. First, as the VIDUS study is not a random sample, our study findings may not be generalizable to PWID at large. Second, the self-reported data may have been affected by responding bias, including socially desirable responding and recall bias. If these sources of bias were present, the true prevalence of risk behaviors assessed would be underestimated, which would bias our findings towards the null. Third, as with all observational research, the estimated relationships between the explanatory variables and assisted injecting may be under the influence of unmeasured confounding, although we sought to address this bias through multivariable adjustment involving key potential demographic, behavioral, social/structural, and environmental confounders.

## Conclusions

In summary, our study demonstrated that rates of assisted injecting have significantly declined among our sample of PWID in Vancouver between 2006 and 2014, which is encouraging as assisted injecting is a major risk factor for a number of negative health consequences. However, rates of assisted injecting remain high, especially among women. We urge policy makers to reconsider the legal framework of existing SIFs to allow assisted injecting as this would reduce a number of harmful consequences for some of the most marginalized and vulnerable PWID.

## Abbreviations

CI: confidence interval
IQR: interquartile range
OR: odds ratio
PO: prescription opioid
PWID: people who inject drugs
VANDU: Vancouver Network of Drug Users
